# Childhood and persistent ADHD symptoms associated with educational failure and long-term occupational disability in adult ADHD

**DOI:** 10.1007/s12402-014-0126-1

**Published:** 2014-02-05

**Authors:** Mats Fredriksen, Alv A. Dahl, Egil W. Martinsen, Ole Klungsoyr, Stephen V. Faraone, Dawn E. Peleikis

**Affiliations:** 1 University of Oslo, 0318 Oslo, Norway; 2 Department of Research, Division of Mental Health and Addiction, Vestfold Hospital Trust, 3101 Tonsberg, Norway; 3 Department of Oncology, Oslo University Hospital, Radiumhospitalet, 0424 Oslo, Norway; 4 Division of Mental Health and Addiction, Oslo University Hospital, 0424 Oslo, Norway; 5 Department of Psychiatry and Behavioral Sciences, SUNY Upstate Medical University, Syracuse, NY 13210 USA; 6 Department of Psychiatry, Grorud Outpatient Clinic, Akershus University Hospital, 1478 Lorenskog, Norway

**Keywords:** Attention deficit, Adult ADHD, School dropout, Comorbidity, Occupational disability

## Abstract

Few studies have examined the impact of childhood attention deficit hyperactivity disorder (ADHD) symptoms on adult ADHD functional outcomes. To address this issue dimensionally, ADHD symptoms in childhood and adulthood and their relation to educational deficits and work disability are studied in a clinical sample of adult patients with previously untreated ADHD. About 250 adults diagnosed systematically with ADHD according to DSM-IV were prospectively recruited. Primary outcomes were high school dropout and being out of the work last year. Childhood ADHD symptoms, sex differences, comorbidities of other mental disorders, and adult ADHD symptoms were examined by historical data, clinician interviews, and questionnaires. High levels of ADHD symptom severity in childhood were related to dropping out of high school [odds ratio (OR) = 3.0], as were higher numbers of hyperactive–impulsive symptoms in childhood. Significantly, more women than men were long-term work disabled (OR = 2.0). After adjusting for age and gender, persisting high levels of ADHD inattention symptoms in adulthood (OR = 2.5), number of comorbid disorders, and particularly anxiety disorders were significantly related to long-term work disability. Childhood hyperactive–impulsive symptoms and overall severity of childhood ADHD symptoms were associated with high school dropout rates; however, persisting ADHD inattention symptoms and comorbid mental disorders in adulthood were more correlated to occupational impairment. These findings underline proposals for studies on early recognition and interventions for ADHD and psychiatric comorbidity. They further suggest that inattentive symptoms be a focus of adult ADHD treatment and that workplace interventions be considered to prevent long-term work disability.

## Introduction

Attention deficit hyperactivity disorder (ADHD) is a childhood-onset neurodevelopmental condition characterized by age-inappropriate levels of inattention, hyperactivity, and impulsivity (H/I) (Kooij et al. 2010). Numerous follow-up studies have confirmed the persistence of ADHD symptoms into adulthood (Barkley et al. 2002; Biederman et al. 2011, 2012; Fayyad et al. 2007; Kessler et al. 2005a, 2006; Lara et al. 2009; Mannuzza et al. 2003; Sandra Kooij et al. 2005). Adolescents and young adults with childhood ADHD have significantly poorer academic achievement and work performance compared with those who did not have ADHD as children (Barkley et al. 2006; Hechtman et al. 1984). As adults, they also have more comorbid mental disorders, including drug and alcohol abuse (Hechtman and Weiss 1986; Biederman et al. 2006). Childhood onset and persistent adult ADHD thus seem to have serious long-term consequences (Able et al. 2007; Adler 2007; De Graaf et al. 2008; Kessler et al. 2005b). Poor academic achievement and long-term work disability may be considered particularly relevant for adult functioning due to their widespread social, financial, and personal consequences (Biederman et al. 2008).

To date, several studies of adult ADHD outcomes have reported on categorical ADHD subtype differences (McGough et al. 2005; Millstein et al. 1997; Murphy et al. 2002; Sprafkin et al. 2007). Sobanski et al. (2008) reported no significant differences between the subtypes concerning functional outcomes; all ADHD subgroups had significantly less education, were more frequently unemployed, and had more life time psychiatric comorbidity compared with non-ADHD controls. Accordingly, Murphy et al. (2002) reported that compared with community controls without ADHD, both the combined ADHD subtype and the inattentive subtype had significantly less education, lower proportion of graduation from college, and they were more likely to have received special education in school. On the other hand, Halmoy et al. (2009) found some differences between the subgroup categories. In their sample, only 24 % of adults with ADHD were gainfully employed, compared with 79 % of population-based controls, and they reported comorbid substance abuse, depression, or anxiety disorders and, belonging to the ADHD-combined subtype, were significantly associated with being out of work. In contrast, Gjervan et al. (2012) found that higher current inattentiveness in adulthood was significantly related to fewer days at work in adults with ADHD.

However, categorical approaches to study subtype have been questioned. A considerable literature shows that DSM-IV childhood ADHD subtypes lack stability over time (Lahey et al. 2005; Nigg et al. 2010). Also, there is an age-dependent tendency for H/I symptoms to decline at a higher rate than inattention (In) symptoms during childhood (Faraone et al. 2006a). Therefore, in the context of predicting adult outcomes, examining the number or accumulated load of In- and H/I-symptoms along with total symptom severity in childhood may be more appropriate than subtype categories (Lahey and Willcutt 2010).

Few studies of patients diagnosed with ADHD in adulthood also assessed ADHD symptoms in childhood. In a prospective, follow-up study of males diagnosed with ADHD in childhood, Mannuzza et al. (2011) reported that a high number of ADHD symptoms in adulthood were associated with increased impairment in adulthood. In a retrospective study, Kessler et al. (2010) found that In-symptoms were more frequently persistent into adulthood than H/I-symptoms; the strongest predictor of ADHD persistence into adulthood was childhood ADHD symptom severity.

Differences between the sexes regarding adult outcomes are of particular interest. Unlike studies of childhood ADHD, which report a greater preponderance of males, gender ratios tend to be equal in studies of adult ADHD (Barkley et al. 2010; Faraone et al. 2006b). Some studies find that more women than men are diagnosed with ADHD in adulthood (Groenewald et al. 2009) and that women are less frequently diagnosed with H/I-symptoms in childhood than males (Rucklidge 2010). Girls with ADHD are also reported to suffer from internalizing symptoms of anxiety and depression and comorbid mental disorders more frequently than boys (Staller and Faraone 2006), although it is questionable whether these differences are maintained in adulthood (Quinn 2008). In a study of adult ADHD, Grevet et al. (2006) found no significant sex differences as regards ADHD subtypes or psychiatric comorbidity. Another study of adult ADHD (Rasmussen and Levander 2009) reported that substance abuse and criminality were more prevalent among men, and affective, eating, and somatization disorders more common among women. However, ADHD symptom severity and subtype did not differ between the sexes. Unlike these results, women were more affected than men on different ADHD scales in another retrospective study (Robison et al. 2008).

In summary, most studies of adult ADHD have studied ADHD symptoms in adulthood, and the reported relations between ADHD symptoms and functional outcomes such as education and work status have not been consistent. Few studies of adult ADHD have specifically examined childhood ADHD symptoms dimensionally, and their ability to predict educational attainment in adults with ADHD (Gau 2011), and there is a sparse and ambiguous literature about predictors of long-term work disability. More research in the area could suggest how to prevent educational failure and occupational impairments and provide directions for further research. Therefore, the aims of our study were to examine whether the number of assessed childhood ADHD symptoms and ADHD symptom severity were associated with lower levels of education and long-term work disability in clinically referred treatment naïve adults with ADHD. Further, we sought to examine whether these associations were moderated by persisting ADHD symptoms in adulthood, gender, and comorbidity.

## Methods

### Site and sampling

The present study is a part of an ongoing prospective observational study of the medical treatment of adults with ADHD at the Outpatient Clinic at the Division of Mental Health and Addiction, Vestfold Hospital Trust, Norway, which is located in the South-Eastern part of Norway, and covers a region of about 250,000 adult inhabitants. Most individuals suspected of adult ADHD within that region are referred to the clinic. Referred patients aged 18–60 years were recruited consecutively. For inclusion, the subjects had to fulfill DSM-IV criteria for ADHD which involve determining the presence of ADHD symptoms during both childhood and adulthood.

The exclusion criteria were any clinically unstable mental disorder that needed immediate treatment, any medical contraindications for stimulant treatment such as hyperthyroidism, cardiovascular diseases, or cardiac arrhythmias, patients having previously tried stimulant medication in adulthood or during the prior 5 years for patients 18 years of age. We also excluded patients with an Intelligence Quotient (IQ) under 70 based on the Wechsler Adult Intelligence Scale IV (Wechsler 2008).

During the ascertainment of participants, 620 referred patients were assessed for eligibility (May 2009–December 2010). The mean age of the females (*n* = 283) was 32.4 (SD = 10.9) years, and for males 31.5 (SD = 10.7) years. By evaluation for inclusion, 262 patients (42 %) were eligible. Twelve did not consent to take part, leaving a total of 250 stimulant naïve adult patients for the study.

### Procedures

#### Psychiatric assessments

To obtain diagnoses, two board-certified psychiatrists examined each patient for inclusion criteria. The ADHD diagnosis was ascertained by a multistage and multisource procedure according to DSM-IV-TR criteria (American Psychiatric Association 2000; Barkley 2008; Faraone et al. 2006b; Haavik et al. 2010) with:

1. *The structured Diagnostic Interview for ADHD in adults, second edition* (*DIVA* 2.0) (Kooij and Francken 2010). To be diagnosed with ADHD, patients must have endorsed at least six out of nine DSM-IV symptoms of inattention and/or hyperactivity/impulsivity in childhood, and currently have at least six out of nine DSM-IV symptoms of inattention and/or hyperactivity/impulsivity for the last 6 months, and describe a chronic course from childhood to adulthood. We also included patients with five out of nine symptom criteria for each symptom domain in adulthood if they had met full symptom criteria in childhood. Patients meeting the lower diagnostic threshold would be diagnosed as ADHD NOS in DSM-IV, but would be diagnosed with full threshold ADHD in DSM-V according to the revised requirements for adult diagnosis (American Psychiatric Association 2013). We required that symptoms currently caused clinically significant impairment in social, academic, or occupational functioning.
2. To examine comorbid mental disorders and whether the ADHD symptoms might be better explained by another psychiatric disorder, *the MINI International Neuropsychiatric Interview Plus* (M.I.N.I.-Plus) was conducted by the clinicians. It is a structured diagnostic interview for DSM-IV Axis I disorders (Sheehan et al. 2002, 1998) for assessing comorbidity.
3. *Supplementary data* to support evidence of childhood symptoms were collected where available from *other informant sources* such as school records, educational psychology services in school, and questionnaires rated by the parents (83 % of the patients), blinded for other informants’ ratings. Collateral information about current symptoms and impairment were also obtained in the majority of cases from a close relative invited to participate during the DIVA interview with the patient.

During a pilot period, 21 adult patients were independently examined by two psychiatrists. For the ADHD DSM-IV diagnosis, *Cohen’s kappa coefficient* was 0.77; *kappa* was 0.88 for ADHD hyperactive–impulsive criteria and 0.70 for inattention criteria. For the clinicians’ assessments of comorbid mental disorders into the applied diagnostic categories of the M.I.N.I.-Plus, measure of agreement showed a *kappa coefficient* of 0.79.

#### Definition and measures of outcome

Our two primary outcome measures were: (1) *Low level of education* defined as *not completed high school* by dropout from or interruption of the expected course of education before ending a secondary school program equivalent to high school including vocational school programs, and (2) *Long*-*term work disability* defined as being out of work in the past year, by being fully out of paid work, ordinary school, or studies due to disability for the last 12 months before enrollment in the study. Data on education were supplemented with historical data collected from school grades, and these measures were ascertained by face-to-face interview with the patients.

Because DSM-IV ‘subtypes’of ADHD have been criticized (Nigg et al. 2010; Willcutt et al. 2012), and the DSM-V renames ‘subtypes’ as ‘presentations,’ we decided to investigate the predictive ability of ADHD symptom dimensions rather than subtypes. Assessments by the DIVA 2.0 allowed for clinician evaluation of symptom criteria of childhood and adulthood separately. Other factors studied in relation to the two main outcome variables were risk to get into fights, antisocial traits, learning difficulties, ratings of child and adult ADHD symptoms, and clinicians’ investigation of current comorbidity.

#### The Wender Utah Rating Scale (WURS)

To identify the severity of retrospective childhood ADHD symptoms, patients rated the Norwegian short version of the WURS-25 (Ward et al. 1993; Wierzbicki 2005), a retrospective dimensional measure of ADHD symptoms which has good psychometric properties (Caci et al. 2010; Fossati et al. 2001; McCann et al. 2000; Retz-Junginger et al. 2003). The WURS-25 items are rated on a 5-point severity scale (score range from 0 to 100). We categorized the scaled ‘WURS-25’ (median = 56, mean = 56, SD = 16.9) into the ‘WURS-25 category’ by quartiles (score <40 or ‘low,’ score 40–70 as ‘moderate,’ and score ≥70 as ‘high’). The WURS subscales for *medical problems* and *school problems* were also examined.

#### Adult ADHD Self-Report Scale version 1.1 (ASRSv.1.1)

Current adult ADHD symptoms present for last 6 months were dimensionally rated by the Norwegian version of the 18 item ASRSv.1.1 (Kessler et al. 2005c). The ASRS covers the nine inattentive criteria and nine hyperactive–impulsive criteria according to DSM-IV (Adler et al. 2006; Kessler et al. 2007; Murphy and Adler 2004). We used the continuous scoring method (Kessler et al. 2005c). Symptoms are self-rated on a 5-point scale of frequency with a score range of 0–72 points for total symptom load. The inattentive and hyperactive–impulsive subscales each have a range of 0–36 points. The 18 item ASRS in our sample showed a *Cronbach’s alpha coefficient* of 0.86; the inattention and hyperactivity–impulsivity subscales had *alpha* values of 0.73 and 0.80, respectively.

#### Additional information

Historical data about pedagogical assistance in primary school, reading or arithmetic problems, grades from school reports, relevant information from other sources on childhood symptoms such as school records and psychological-pedagogic services records, were also collected systematically. Physical examination was performed by the patients’ regular physician within the past 3 months to exclude somatic diseases, and data from medical records of previous medication were collected.

To evaluate intellectual ability, all patients with school grades below average were screened by the Hayes Ability Screening Index (HASI) (Hayes 2000; Sondenaa et al. 2011). Exclusions of those with an IQ under 70 were based on the Wechsler Adult Intelligence Scale IV (Wechsler 2008), which was performed when for HASI score less than or equal to 85.

### Ethics

The study was approved by The Regional Committee for Medical and Health Research Ethics of South-Eastern Norway and The Norwegian Social Science Data Services and has therefore been performed in accordance with the ethical standards in the 1964 Declaration of Helsinki and later amendments. After a complete description of the study was provided to the subjects, all participants gave written informed consent prior to their inclusion in the study.

### Statistical methods

All statistical analyses were carried out by The PASW statistics (version 17) for Windows package. Data from the clinician administered MINI on comorbid mental disorders are aggregated within each diagnostic category presented such as any panic disorder or obsessive compulsive disorder are represented as any anxiety disorder. The data were initially analyzed by descriptive methods. On the group level, categorical variables were analyzed using the chi-square test and stratified in order to explore sex effects (Grevet et al. 2006). Testing differences in the continuous outcomes was done with *t* tests when the assumption of normal distribution was met, and otherwise with nonparametric tests. The level of significance in the univariate analyses was a priori set at *p* < 0.01 due to numbers of tests planned. All tests were two sided. The continuous scores of the WURS-25, and ASRS inattention subscale were categorized into *low*, *moderate*, and *high* level by the quartiles to get a more clinically relevant representation.

Analyses of the dependent categorical factor ‘not completed high school’ were adjusted for gender, but not the ‘age’-factor because predictors should antecede the predicted factor. The work status category analyses was conducted adjusted for both age and gender. The specified independent variables found to have significant associations to the dependent variable were entered into logistic regression models initially unadjusted one at a time and were finally adjusted by entering age and gender together. Corresponding odds ratios (ORs) and their 95 % confidence interval (95 % CI) were estimated as measure of strength of associations, and the level of two-tailed significance was set at *p* < 0.05.

## Results

### Sociodemographics and clinical characteristics by gender

A total of 250 patients participated. Their mean age was 32.6 (SD = 9.8) years, and 52 % were females. Only five patients (2 %) had received an ADHD diagnosis before 18 years of age. None had previously received any targeted ADHD treatment or stimulant medication. Table 1 presents socio-demographic and clinical characteristics for all patients and stratified by gender. The females had a significant higher age at referral to the clinic than the males.

**Table 1:** Sociodemographic and clinical characteristics by sexes

	Total sample	Females	Males	p*
	(*n* = 250)	(*n* = 129)	(*n* = 121)	–
Age, mean (SD)^b^	32.6 (9.8)	34.1 (9.8)	30.7 (9.5)	**<0.01**
School variables, *n* (%)				
Not completed high school	140 (56)	67 (52)	73 (60)	0.18
Number of years with education, mean (SD)	11.0 (2.3)	11.3 (2.4)	10.8 (2.1)	0.12
Behavioral risk to get into fights^a^	108 (43)	47 (41)	61 (56)	**0.03**
Educational psychology services	73 (29)	26 (20)	47 (39)	**<0.01**
Lexical or arithmetical skill problems	95 (38)	46 (36)	49 (41)	0.43
Childhood symptoms				
Wender Utah Rating Scale^b^, mean (SD)				
WURS medical problems	5.0 (4.4)	5.8 (4.5)	4.0 (4.1)	**<0.01**
WURS school-related problems	17.9 (7.6)	16.0 (7.2)	19.8 (7.5)	**<0.01**
WURS-25	56.2 (16.8)	55.9 (17.7)	56.2 (16.1)	0.90
WURS-25 category, *n* (%)				
Low (<40)	40 (16)	26 (20)	14 (12)	**0.05**
Moderate (40–70)	152 (61)	70 (54)	82 (69)	–
High (≥70)	56 (23)	33 (26)	23 (19)	–
Number of ADHD symptoms in childhood (by DIVA^c^), mean (SD)				
Total number of symptoms	11.8 (3.1)	11.5 (3.2)	12.2 (2.9)	0.07
Inattention symptoms	6.6 (1.5)	6.5 (1.4)	6.7 (1.6)	0.25
Hyperactivity–impulsivity symptoms	5.2 (2.4)	5.0 (2.5)	5.5 (2.3)	0.11
Adult factors				
Occupational status at inclusion, *n* (%)				
In paid work or study	111 (44)	47 (36)	64 (53)	**<0.01**
Disability or rehabilitation pension	124 (50)	75 (58)	49 (41)	**<0.01**
Unemployed	15 (6)	7 (5)	8 (6)	0.69
Work disabled last year	128 (51)	77 (60)	51 (42)	**<0.01**
Comorbid mental disorder^a^, *n* (%)				
Any comorbid mental disorder	188 (75)	96 (74)	92 (76)	0.77
Major depressive episode	59 (24)	32 (25)	27 (22)	0.64
Bipolar disorder	39 (16)	20 (16)	19 (16)	0.97
Anxiety disorder (last year)	136 (54)	77 (60)	59 (49)	0.08
Alcohol-use disorder (last year)	38 (15)	16 (12)	22 (18)	0.20
Drug-use disorder (last year)	36 (14)	13 (10)	23 (19)	**0.04**
Antisocial behavior	42 (17)	10 (8)	32 (27)	**<0.01**
Number of comorbid disorders^c^, mean (SD)	1.5 (1.2)	1.5 (1.2)	1.5 (1.2)	0.78
Number of ADHD symptoms in adulthood (by DIVA^c^), mean (SD)				
Total number of symptoms (0–18)	12.0 (3.2)	12.5 (3.0)	11.5 (3.3)	**<0.01**
Inattention symptoms (0–9)	6.8 (1.7)	7.1 (1.3)	6.5 (2.0)	**<0.01**
Hyperactivity–impulsivity symptoms (0–9)	5.2 (2.3)	5.4 (2.3)	5.0 (2.3)	0.16
Adult ADHD Self-Report Scale (ASRSv1.1), mean (SD)				
ASRS full scale (0–72)	50.5 (10.2)	51.9 (9.3)	48.8 (10.9)	**0.01**
Inattention subscale (0–36)	26.9 (5.3)	27.7 (4.6)	26.0 (5.6)	**<0.01**
Hyperactivity–impulsivity subscale (0–36)	23.6 (6.6)	24.2 (6.3)	22.8 (7.0)	0.09
Psychopharmacological medication last year (missing = 7), *n*(%)	173 (71)	96 (77)	77 (65)	**0.05**

* Statistically significant and near-significant *p*-values are in bold

^a^By the Mini International Neuropsychiatric Interview (M.I.N.I.), life time if not specified

^b^Severity of childhood symptoms rated by the Wender Utah Rating Scale, WURS-25 category by the quartiles: WURS-25 score <40 → 0 (ref.)

^c^The structured Diagnostic Interview for ADHD in adults, second edition (DIVA 2.0); number of symptom criteria met in childhood and adulthood assessed separately by the clinicians

### Sex differences in childhood characteristics (Table 1)

Many patients reported problems with getting involved in fights with peers in childhood (43 %) indicating impulsive behavior, a tendency that was significantly more common in males than females (Table 1). A substantial proportion (38 %) of all patients had reading or arithmetic skill problems in primary school. These problems were evenly distributed between the sexes, but significantly more males had received assessments by educational psychology services during primary school (39 vs. 20 %, *p* < 0.01). Regarding severity of childhood symptoms by the WURS, males reported less medical problems in childhood (*p* < 0.01), and more school-related problems (*p* < 0.01) compared to females, but no significant differences in overall childhood ADHD symptom scores were observed between the sexes. Likewise, we did not found any significant sex differences in the number of DIVA-assessed childhood ADHD symptoms.

### Sex differences in comorbidity, adult symptoms, education, and work status

A large proportion of the total sample had at least one comorbid mental disorder (75 %), and 55 % had two or more such disorders (not shown in the table). Except for more males having antisocial behavior, we found no significant differences between the sexes (Table 1). A significant proportion of the patients in our sample used or had used a medicine for anxiety or depression in the past year (71 %). There was a tendency for more women than men to use such medications (77 vs. 65 %, *p* < 0.05) which consisted mainly of antidepressants (>90 %), to a lesser degree anxiolytics and sedatives. Such medication was related to higher number of comorbid disorders (*p* < 0.01) (not shown in the tables).

The number of clinician assessed ADHD symptoms in adulthood by the DIVA was significantly higher for females (*p* < 0.01), particularly for inattentive symptoms (*p* < 0.01). Self-reported current ADHD symptoms differed correspondingly with a significant tendency for more frequent symptoms reported by females than males on the ASRS full scale (*p* < 0.01) and the inattentive subscale (*p* < 0.01).

A considerable proportion of the sample (56 %) had not completed a secondary school program equivalent to high school; no significant difference between the sexes was observed. However, more males (53 %) than females (36 %) were in paid work or ordinary studies at enrollment in the study (*p* < 0.01), and more females (58 %) were reported with disability or rehabilitation pension than males (41 %) (*p* < 0.01) (Table 1). This sex difference was also reflected in the long-term outcome of being disabled for work in the past year (60 % of women vs. 42 % of men, *p* < 0.01).

### Factors associated with high school noncompletion (Table 3)

Mean age at inclusion into the study was higher among those who had completed high school (35.1, SD = 9.7 years vs. 30.2, SD = 9.4 years, *p* < 0.01) (Table 2). Those who did not complete high school more often reported *risk to get into fights* *with peers*, and a larger proportion had received assessment by educational psychology services during primary school. The noncompleters had significantly higher scores on overall child ADHD symptom severity (WURS-25, *p* < 0.01) and had been assessed by the clinicians as having significantly more DSM-IV ADHD symptoms and H/I-symptoms in childhood (DIVA) (*p* = 0.01). No significant differences were found for comorbid mental disorders or ADHD symptoms in adulthood (ASRS) between the two education categories.

**Table 2:** Characteristics and symptoms by high school dropout and long-term work disability

	High school dropout (*n* = 140)	Completed high school (*n* = 110)	*p* ^a^	Work disabled (*n* = 128)	Employable last year (*n* = 122)	*p* ^a^
Age, mean (SD)^b^	30.2 (9.4)	35.1 (9.7)	**<0.01**	34.3 (9.8)	30.6 (9.5)	**<0.01**
Education, *n* (%)						
Risk to get into fights^c^	68 (56)	40 (39)	**0.01**	58 (50)	50 (45)	0.42
Educational psychology services	50 (36)	23 (21)	**0.01**	34 (27)	32 (27)	0.98
Lexical or arithmetical skill problems	55 (40)	40 (37)	0.64	53 (41)	42 (35)	0.30
Not completed high school	–	–	–	76 (59)	64 (53)	0.27
Number of years with education, mean (SD)^b^	9.4 (0.7)	13.1 (2.0)	**<0.01**	10.8 (2.0)	11.3 (2.6)	0.08
The Wender Utah Rating Scale^c^, mean (SD)^b^						
WURS medical problems	5.0 (4.7)	5.0 (4.1)	0.96	5.5 (4.8)	4.4 (3.9)	**0.04**
WURS school-related problems	18.6 (7.5)	16.8 (7.7)	0.07	17.8 (7.6)	17.8 (7.6)	0.95
WURS-25	58.7 (18.0)	52.7 (14.9)	**<0.01**	57.0 (16.4)	55.0 (17.5)	0.34
WURS-25 category^a^, *n* (%)						
Low (<40)	20 (15)	20 (18)	**<0.01**	17 (13)	23 (19)	0.48
Moderate (40–70)	76 (55)	76 (69)	–	80 (63)	72 (60)	–
High (≥70)	42 (30)	14 (13)	–	30 (24)	26 (22)	–
Number of ADHD symptoms in childhood^d^, mean (SD)^b^						
Total number	12.4 (2.9)	11.1 (3.2)	**<0.01**	11.5 (3.2)	12.1 (3.0)	0.11
Inattention	6.8 (1.4)	6.4 (1.6)	**0.04**	6.5 (1.5)	6.6 (1.5)	0.54
Hyperactivity–impulsivity	5.6 (2.3)	4.7 (2.5)	**<0.01**	5.0 (2.6)	5.5 (2.1)	0.09
Comorbid disorders^e^, *n* (%)						
Depressive disorder	13 (9)	18 (16)	0.09	15 (23)	16 (10)	0.72
Bipolar disorder	21 (15)	18 (16)	0.77	25 (20)	14 (12)	0.08
Anxiety disorder (last year)	79 (56)	57 (52)	0.47	82 (64)	54 (44)	**<0.01**
Alcohol-use disorder (last year)	26 (19)	12 (11)	0.09	20 (16)	18 (15)	0.85
Drug-use disorder (last year)	23 (16)	13 (12)	0.30	20 (16)	16 (13)	0.57
Antisocial or conduct behavior	25 (18)	17 (16)	0.60	23 (18)	19 (16)	0.59
Number of comorbid disorders^d^, mean (SD)^b^	1.5 (1.1)	1.4 (1.2)	0.36	1.8 (1.2)	1.2 (1.1)	**<0.01**
Number of ADHD symptoms in adulthood^d^, mean (SD)^b^						
Total number of symptoms (0–18)	12.2 (3.2)	11.8 (3.1)	0.28	12.2 (3.1)	11.8 (3.4)	0.35
Inattention (0–9)	7.0 (1.8)	6.6 (1.6)	0.07	7.0 (1.4)	6.6 (1.9)	**0.04**
Hyperactivity–impulsivity (0–9)	5.3 (2.3)	5.2 (2.2)	0.84	5.2 (2.3)	5.3 (2.2)	0.84
Adult ADHD Self-Report Scale, mean (SD)^b^						
ASRS full scale (0–72)	51.0 (10.7)	49.7 (9.4)	0.31	51.2 (10.2)	49.6 (10.1)	0.21
Inattention subscale (0–36)	27.3 (5.5)	26.4 (4.9)	0.14	27.4 (4.8)	26.0 (5.6)	**0.01**
Hyperactivity–impulsivity subscale (0–36)	23.6 (6.8)	23.3 (6.6)	0.70	23.4 (7.0)	23.5 (6.4)	0.90
Psychopharmaca last year, *n* (%) (not ADHD-medication) (missing = 7)	91 (68)	82 (75)	0.21	103 (82)	70 (60)	**<0.01**

Patients classified by *completed high school* or not (lower education) and *being out of work or study last year* by disability

^a^From Pearson chi-square, 2-sided test if not otherwise specified. Statistically significant or near-significant *p*-values are displayed in bold

^b^ *t* test, independent groups, 2-sided if not otherwise specified

^c^Severity of childhood symptoms rated by the Wender. Utah Rating Scale WURS-25 category by the quartiles: WURS-25 score <40 → 0 (low = ref.)

^d^The structured diagnostic interview for ADHD in adults, second edition (DIVA 2.0), number of symptom criteria met in childhood and adulthood assessed separately

^e^The Mini International Nevropsychiatric Interview (M.I.N.I.) plus version for DSM-IV, lifetime if not otherwise specified

Previous assessment by *educational psychology services* during (primary) school and *risk to get into fights* with peers during childhood was significantly associated with *likelihood of not completed high school* when entered one at a time in the regression analyses. After adjusting for sex, these associations remained significant (*p* < 0.05).

The WURS-25 category of ADHD symptom severity ‘high’ (score ≥70) predicted noncompletion of high school with OR = 3.0. This indicates a threefold increased risk of not attaining upper secondary education. Higher numbers of child ADHD H/I-symptoms also remained significantly related to noncompletion of high school after adjustment for sex (*p* < 0.01). To control for confounding of disruptive behavior, the number of H/I-symptoms in childhood was entered into the regression model adjusted for *risk to get into fights* with peers (M.I.N.I.-Plus). With this adjustment, the association to noncompletion then became statistically nonsignificant. However, alternatively adjusting for occurrence of *antisocial*-*/conduct disorder symptoms* (M.I.N.I.-Plus), the number of H/I-childhood symptoms maintained statistically significant (*p* < 0.01) as a predictor of high school dropout (not shown in the tables).

### Factors associated with work status

The majority of the sample was not in paid work or study (56 %) at the time of referral, and 51 % had not been in paid work or study in the past year due to disability. Half of the sample also received a disability or rehabilitation pension, reflecting long-term work disability (Table 1). A significantly larger proportion of females (60 %) than males (42 %) had not been in paid work or study last year (Table 1). The mean age at referral of those *out of work or study* was significantly higher than among those working (Table 2). Overall childhood severity scores by the WURS-25 showed no difference between employed and work-disabled patients.

Those who were out of work last year had significantly more comorbid disorders (*p* < 0.01), particularly anxiety disorders in both sexes, and antisocial or conduct disorder among females (8 vs. 0 %, *p* < 0.01; data not in the tables). A significant proportion of the patients in the group of long-term work disability used or had used a medicine for anxiety or depression in the past year (82 %, *p* < 0.01). Those *out of work or study last year* also had significantly higher ASRS inattention scores compared with those in work or study (*p* < 0.01). The patients who were not working also showed a tendency toward having more inattentive symptoms in adulthood (*p* = 0.04) assessed by DIVA (Table 2).

### Predicting long-term work disability

Higher age at entering the study was significantly related to higher *likelihood of being out of work last year*, and females were more at risk with age adjusted OR = 1.8 in the logistic regression analyses. When adjusting for age and sex, higher numbers of persistent ADHD In-symptoms in adulthood assessed by the DIVA with OR = 1.2 (Table 3), and level of persistent ADHD inattentive symptoms in adulthood measured by the ASRS inattention score with adjusted OR = 1.1 (not shown in the tables), and of the ASRS inattention *high score category* with adjusted OR = 2.5, by categories of the quartiles score(*low* <24, *high* ≥31) were related to increased likelihood of *being out of work last year* (Table 3). Also, higher *number of comorbid mental disorders* (MINI) remained significantly related to *not being in work last year* with adjusted OR = 1.6 (Table 3). A significant inter-correlation between number of comorbid disorders and ADHD symptoms (ASRS total score) and inattention symptoms (ASRS inattention score) was found (*Spearman’s rho* = 0.22, *p* < 0.01 and *ρ* = 0.19, *p* < 0.01, respectively). The statistically significant associations found between the ASRS inattention *high score category* and the dependent variable of work disability (Table 3) disappeared when combined with number of comorbid disorders in the regression model (data not shown).

**Table 3:** Prediction of adult functional outcome unadjusted and adjusted for age and sex by logistic regression analyses

(*n* = 250)	Low education		Work disability	
	Likelihood of ‘not completed high school’^a^		Likelihood of ‘being out of work last year’^a^	
	Unadjusted OR (CI)	Adjusted OR (CI)	Unadjusted OR (CI)	Adjusted OR (CI)
Age (years)	Not in equation	Not in equation	1.04 (1.01–1.07)**	1.04 (1.01–1.06)**
Sex				
Male	1.0	Ref.	1.0	Ref.
Female	0.7 (0.43–1.17)	0.7 (0.43–1.17)	2.0 (1.23–3.36)**	1.8 (1.10–3.08)*
Childhood factors				
Educational psychology services	2.1 (1.18–3.74)*	0.5 (0.28–0.90)*	1.0 (0.56–1.68)	Not in equation
Risk to get into fights (M.I.N.I.^b^)	2.0 (1.18–3.43)**	1.9 (1.14–3.34)*	0.8 (0.48–1.36)	–
WURS-25 category^e^				
Low category (<40)	1.0	Ref.	1.0	–
Moderate (40–70)	1.0 (0.50–2.01)	0.9 (0.46–1.88)	1.5 (0.74–3.03)	–
High (≥70)	3.0 (1.26–7.13)**	3.0 (1.24–7.05)**	1.5 (0.69–3.54)	–
WURS medical	1.0 (0.94–1.06)	Not in equation	1.1 (1.002–1.13)*	1.0 (0.99–1.11)
Number of ADHD hyperactive–impulsive symptoms in childhood^d^	1.2 (1.05–1.30)**	1.1 (1.01–1.26)*	0.9 (0.82–1.02)	Not in equation
Adult factors				
Comorbid disorders^b^	Not in equation	Not in equation	1.6 (1.28–2.04)***	1.6 (1.27–2.06)***
Number of persistent ADHD Inattention symptoms in adulthood^d^	–	–	1.2 (1.005–1.36)*	1.2 (1.002–1.38)*
ASRS inattention score^e^				
Low category (<24)	–	–	1.0	Ref.
Moderate (24–31)	–	–	1.5 (0.86–2.77)	1.5 (0.81–2.73)
High (≥31)	–	–	2.7 (1.26–5.59)**	2.5 (1.16–5.38)*

Statistically significant estimates of OR are in bold

* *p* < 0.05, ** *p* < 0.01, *** *p* < 0.001, OR (95 % CI), logistic regression, sig. two-tailed

^a^Logistic regression, specified variables entered in the equation, unadjusted odds ratio (OR) estimated by entering factors one at a time in the equation. Probability stepwise for entry 0.05, removal 0.10. Education status is adjusted only for gender, and work status is adjusted for age and gender

^b^Number of disorders by the Mini International Nevropsychiatric Interview (M.I.N.I.) plus version for DSM-IV

^c^WURS—25 category: WURS—25 score <40 → low (ref.), score between 40 and 70 → moderate and score ≥70 → high

^d^Number of ADHD symptoms by DIVA (DSM-IV ADHD criteria) of childhood and adulthood assessed separately

^e^Adult ADHD Self-report Scale, subscale of 9 ADHD inattentive symptoms (score range 0–36); inattention category by quartiles: score <24 → low (ref.)

## Discussion

### Main findings

This study examined adult ADHD outcomes of school failure and long-term work disability in dimensional relation to previous and persistent ADHD symptoms and comorbidity for both genders. Patients who had more hyperactive and impulsive symptoms and total ADHD symptoms in childhood had a significantly shorter duration of education and an increased risk of dropout from high school. No significant sex differences were observed concerning these educational outcomes. Work status was weakly related to differences in ADHD characteristics in childhood. In contrast, persistent inattentive ADHD symptoms in adulthood, as well as the number of comorbid mental disorders in adulthood, were significantly related to an increased risk of long-term work disability. Significantly more women than men were long-term work-disabled.

### Childhood hyperactivity–impulsivity predicted high school drop-out

Longitudinal studies of children with follow-up in youth and young adulthood have found adverse educational outcomes associated with the severity of childhood ADHD symptoms (Barbaresi et al. 2007; Barkley et al. 1990). Youths with high levels of hyperactive–impulsive symptoms may be more likely to experience negative feedback in school or to make impulsive decisions about discontinuation, than those with fewer symptoms in this domain. However, in contrast to our study, which found that hyperactive–impulsive symptoms, but not conduct disorder, predicted high school dropout, prior studies found that conduct disorder was the best predictor of high school dropout (Barkley et al. 1990; Breslau et al. 2011). A longitudinal study did not find such an association (Trampush et al. 2009), but lower IQ, reading ability, socioeconomic status, frequent marijuana use, and limited paternal contact significantly differentiated school dropouts from graduates. Our finding of hyperactivity–impulsivity in childhood as a predictor of school failure was modified if adjusted for *risk to get into fights* with peers; and this association became statistically nonsignificant. However, when we adjusted for occurrence of *antisocial*-*conduct behavior* (M.I.N.I.-Plus), the hyperactive–impulsive childhood symptoms remained a statistically significant predictor of high school dropout (not shown in the tables), indicating this association was partially independent of antisocial behaviors.

### Persistent inattentive symptoms related to work disability

While number and severity of ADHD symptoms in childhood were related to lower educational outcomes, these factors seemed not to influence on the outcome of being *out of work last year*. This is in accordance with a report of weak correlations between specific ADHD symptoms and adult impairment (Gordon et al. 2006). However, others found a statistically significant relationship between childhood ADHD symptoms and impairment in adulthood when impairment in broader functional domains was evaluated (Barkley et al. 2006; Kessler et al. 2010; Mannuzza et al. 2011).

We found adult inattentive symptoms to be significantly related to *bei*ng *out of work last year*. Several studies have reported decreases in hyperactive/impulsive symptoms by age, and persistence of inattentive symptoms into adulthood (Faraone et al. 2006a). Gjervan et al. (2012) also found that current inattentiveness in adults was significantly related to fewer days in ordinary work during the last year. Also, according to other authors, the hyperactive/impulsive subtype is less common in adult ADHD (Sobanski et al. 2008), and the inattentive symptom cluster has been reported to be more disabling in ADHD adults (Stavro et al. 2007).

### Educational and vocational deficits and sex differences

Almost twice as many (56 %) as in the general Norwegian population (33 %) of patients in our total sample had not completed high school (Falch et al. 2010), and more than half of the patients had not attained a suitable education for the labor market. However, we did not find any significant association between educational failure and long-term work disability. This may be explained partially by existing needs for unskilled workers and employment measures for unskilled or uneducated workers.

The majority of participants were not employed at the time of referral, and half of them had not been in ordinary work or study during the past year due to disability. These disability rates are very high when compared with the known nonworking rate of 18 % for the Norwegian population (Statistics Norway 2012). We found higher nonworking rates in our sample than reported in some other studies of adults with ADHD (Able et al. 2007; Sobanski et al. 2007), although similar proportions were reported in studies by Halmoy et al. (2009) and Gjervan et al. (2012).

Almost twice as many women than men reported long-term unemployment or being fully out of work last year due to disability; this gender difference remained statistically significant when adjusted for age and comorbidity (Table 3). This finding could not entirely be explained by a general trend in the Norwegian population of more women than men working parttime, nor by mental disorders being more common among women (Statistics Norway 2012), since we compared only those fully out of work, and adjusted for psychiatric comorbidity. This significant sex difference thus could indicate women to be more susceptible than men in a vocational context to the disabling consequences of ADHD, or that women are more prone to get work environments particularly less compatible with ADHD. This raises unresolved questions about unfavorable environmental work place factors, and proposals for counseling or facilitation.

### Comorbidity and functional outcomes

Adult comorbidity was significantly related to long-term work disability in our sample, and for both sexes. A large proportion of our sample had psychiatric comorbidity (75 %) in accordance with reported prevalences from prior studies of adult ADHD. The total number of comorbid disorders did not differ significantly between the sexes or between those who did and did not complete high school. Of adult factors with significant relation to long-term work outcome, number of comorbid disorders correlated marginally with selected childhood factors and thus may reflect an independent factor of child ADHD symptoms.

Some studies have shown worse outcome in childhood ADHD with co-occurring psychopathology (Pliszka 1998; Wilens et al. 2002), and similar results have been found for adults with ADHD (McGough et al. 2005; Mick et al. 2008; Weiss et al. 2010). There is a significant literature showing that psychiatric morbidity and anxiety disorders in adults are associated with long-term work disability (Lorant et al. 2003; Sareen et al. 2006; Virtanen et al. 2011). A significant proportion of the patients in our sample used or had used a medicine for anxiety or depression last year, and such medication use was associated with long-term work disability. This was expected since those on any medication had a higher number of comorbid disorders. Lack of medical treatment of comorbidity therefore was unlikely a significantly confounding factor for these worse outcomes, but the issue of ineffective treatment is still not accounted for.

### Strengths and limitations

Our study has several strengths. A novel contribution of the study is the dimensional examination of whether level of disability in adulthood is related to number of childhood criteria met. The sample comprised treatment naïve, adult ADHD patients and represented a wide age-span, both genders and comorbid conditions. We had historical data from school and consultations with former educational psychology services and health care services data. Collateral independent information was collected for the majority of the patients to ascertain childhood data. All patients in the present study were examined with structured diagnostic interviews by trained clinicians, and evaluations were not based on self-reported questionnaires or retrospective data only.

A large proportion of the patients were previously undiagnosed concerning ADHD, though many had been recognized with learning difficulties or behavioral problems in childhood. Gjervan et al. (2012) from Norway reported a higher proportion diagnosed by the age of 18 in their sample (23/149), but Bejerot et al. (2010) from Sweden reported fewer (1/214) with prior diagnosis, as they recruited patients for treatment of firsttime referred adults with ADHD. Some selection bias that may have contributed to our study is the inclusion criteria of being previously unmedicated, and the fact that more than half of the patients were aged 30 years or older.

However, our findings have to be viewed within the limitations of the design and methods applied. It is a cross-sectional study based on data from a clinical sample. Our findings of statistically significant associations do not imply causal relationships. Furthermore, investigators were not blind to the participants’ diagnostic status, which could have influenced their assessments. However, the data from parents and patients were collected independently; parents did not know the ratings of the patients and vice versa. Still, retrospective data are possibly distorted by current symptoms, which may bias estimates of association.

The clinical variables are not presented together in the regression model due to significant inter-correlation. Effects of multicollinearity were expected to occur in the multivariate analysis, and thus, comorbidity as a confounder could not be fully accounted for.

## Conclusions and implications

Severity of ADHD symptoms and a high load of child hyperactive–impulsive symptoms in childhood were associated with dropout from school and fewer years of attained education, indicating an increased risk for unfavorable educational outcomes related to these symptoms in childhood and adolescence. Persistence of more inattentive symptoms in adulthood was associated with greater occupational impairment, and additional adult comorbidity was a major predictor of long-term work disability.

Our findings emphasize the serious consequences of ADHD in childhood and adulthood in terms of functional outcomes and may suggest that early recognition and intervention for ADHD and comorbid mental disorders are of importance to improve the long-term outcome for ADHD patients. Our work further emphasizes the importance of addressing inattentive symptoms in the treatment of adult ADHD and calls for adequate workplace measures to prevent long-term work disability.
